# Long‐term programming effects on blood pressure following gestational exposure to the *I*_Kr_ blocker Dofetilide

**DOI:** 10.14814/phy2.13621

**Published:** 2018-03-04

**Authors:** Louise Prestipino, Jaimie W. Polson, Elisabeth Brolin, Helen E. Ritchie

**Affiliations:** ^1^ School of Medical Sciences and Bosch Institute Sydney Medical School The University of Sydney Sydney NSW Australia

**Keywords:** embryonic bradycardia, programmed hypertension, stress

## Abstract

A slow embryonic heart rate in early‐mid gestation is associated with increased risk of embryonic death and malformation, however, the long‐term consequences remain unknown. We administered Dofetilide (Dof, 2.5 mg/kg), a drug that produces embryo‐specific bradycardia, to pregnant rats from gestational days 11–14. Embryonic heart rate and rhythm were determined using embryo culture. Cardiovascular function was assessed in surviving adult offspring at rest, during acute psychological stress (air jet stress, AJS), and after 7 days of repeated AJS. Dof reduced embryonic HR by 40% for ~8 h on each of the treatment days. On postnatal day 3, Dof offspring were ~10% smaller. Blood pressure was elevated in adult Dof rats (systolic blood pressure, night: 103.8 ± 3.9 vs. 111.2 ± 3.0 mmHg, *P* = 0.01). While the pressor response to AJS was similar in both groups (control 17.7 ± 3.4; Dof 18.9 ± 0.9 mmHg, *P* = 0.74), after 7 days repeated AJS, clear habituation was present in control (*P* = 0.0001) but not Dof offspring (*P* = 0.48). Only Dof offspring showed a small increase in resting blood pressure after 7 days repeated stress (+3.9 ± 1.7 mmHg, *P* = 0.05). The results indicate that embryonic bradycardia programs hypertension and impaired stress adaptation, and have implications for the maternal use of cardioactive drugs during pregnancy.

## Introduction

There is now robust evidence from human and animal studies that a range of gestational insults impact on fetal growth and increase the risk of cardiovascular diseases in the adult offspring in a process known as developmental programming (Barker 1995; Yeung et al. 2014; Burton et al. 2016). Traditionally, these insults include maternal dietary manipulations, maternal psychological stress, or compromises to fetal perfusion. While reductions in uterine and placental blood flow have been well studied in programming models of preeclampsia, placental insufficiency, and uterine artery ligation (Hutter et al. 2010), potential consequences of reduced postplacental perfusion, for example, following fetal bradycardia (Olgan et al. 2014) are largely unknown. Fetal bradycardia may manifest as a result of congenital channelopathies, maternal autoantibodies, or following exposure to cardioactive drugs (Strasburger and Wakai 2010; Nilsson et al. 2013; Nilsson and Webster 2014).

A number of studies have shown that fetal bradycardia is associated with a high risk of first trimester spontaneous abortion (Doubilet and Benson 2005; Arleo and Troiano 2011) and birth defects (Webster et al. 2014). Thus, pharmaceutical medications that have the capacity to alter cardiac ion channel activity, including class III antiarrhythmics, antidepressants, antipsychotics, macrolide antibiotics, certain antihistamines, and anticonvulsants, are tested for safe use in pregnancy (Webster et al. 2014). However, the endpoints for these studies are structural malformations or embryonic death. No studies are routinely performed to evaluate the long‐term consequences of prenatal exposure to these drugs in surviving, seemingly healthy embryos.

Dofetilide (Dof) is a class III antiarrhythmic drug (*I*_Kr_ channel blocker) that is used in the USA for the treatment of atrial arrhythmias. Dof is known to produce serious side effects, including Torsades de pointes, however, it may be prescribed to pregnant women where the benefit to the patient justifies the risk to her offspring (Joglar and Page 2014). In rodents, experimental evidence indicates that Dof acts specifically on the fetal heart from gestational days GD10–GD14 (Ritchie et al. 2013; Webster et al. 2014). After this time, the channel profile in the heart changes (Davies et al. 1996) and the drug no longer has an effect (Abrahamsson et al. 1994; Nilsson et al. 2013). Therefore, the effect on fetal development can be investigated in a more focused way without having to consider adverse effects in pregnant rats. We have previously shown that Dof induces embryonic bradycardia and/or arrhythmia both in vivo and in vitro (Ritchie et al. 2013, 2015) but the possible long‐term effects on the offspring have not been studied. In this paper, we report on the impact of prenatal exposure to low dose Dof on cardiovascular function in the adult offspring. We hypothesize that prenatal exposure to low‐dose Dof will produce fetal bradycardia and a prohypertensive phenotype in the adult offspring. The results have implications for the use of drugs that induce embryonic bradycardia during pregnancy.

## Materials and Methods

### Ethical approval

All experiments were approved by The Animals Ethical Review Committee of the University of Sydney and performed in accordance with the NSW Animal Research Act (1985) and its associated Regulations, the 8th Edition Australian code for the care and use of animals for scientific purposes of the National Health and Medical Research Council of Australia (2013) and the Australian Code for the Responsible Conduct of Research (2007). Sprague–Dawley rats (Animal Resources Centre, Murdoch, Western Australia) were group housed under a 12:12 h light/dark cycle at 22–26°C and 40–60% humidity with ad libitum access to standard laboratory chow and water.

### Gestational administration of Dofetilide

Female rats (250–350 g) were mated by placing a male in the cage overnight. Mating was confirmed by a vaginal swab positive for sperm. Thirty nine pregnant rats were dosed by gavage with 2.5 mg/kg Dof (Pfizer, Central Research, Sandwich, UK) or water daily from GD11 to GD14. Twenty four dams were used for embryo culture and 15 dams used for telemetry studies. Using high frequency ultrasound, we have previously observed that this Dof dose is associated with a ~60% decrease in embryonic HR on GD11 and ~20% decrease on GD13, followed by recovery 24 h later (Ritchie et al. 2015). Higher daily doses were associated with significant embryonic death.

### Measurement of heart rate in embryo culture

In the first cohort of rats, on each day (GD11‐GD14), at 2 or 9 h after dosing with Dof or water, 2–5 pregnant rats (a total of 11 control and 13 Dof dams) were euthanized by CO_2_ inhalation followed by cervical dislocation. These time points reflect the peak effect and expected postrecovery period (Smith et al. 1992). Embryos were rapidly extracted and prepared for in vitro culture as described previously (Ritchie et al. 2013). Briefly, the intact uterus was rinsed with phosphate‐buffered saline and implantations were counted and removed. The decidua and the Reichert's membrane were removed using forceps under a dissecting microscope. An incision was made in the yolk sac, avoiding major blood vessels. The embryo was then pushed through the incision and the amnion was opened. Embryos (minimum 5 per dam) were rapidly extracted and placed in a culture bottle containing 2.5 mL of Dulbecco's modified Eagle's medium (Sigma‐Aldrich, St. Louis, MO) and continuously gassed with 95% O_2_ and 5% CO_2_ at 37°C. The culture bottles were placed in a rotating culture system (B.T.C. Engineering, Milton, Cambridge, UK) and rotated 30 times per minute. After 15‐min equilibration, the embryo cultures were placed on a heated stage under a dissecting microscope (Leica M420 Leica Microsystems Ltd, Heerbrugg, Switzerland) fitted with a video recorder (Olympus DP70, Olympus Australia, Pty, Melbourne, Australia). The embryonic heart was video recorded for 20 sec (30 frames.s^−1^). The atria and ventricles were identified on the video and analyzed for contraction frequency and rhythm using custom‐made software developed at the University of Uppsala (Ritchie et al. 2015). The researchers were blinded to group identity during the analysis, but not the acquisition of the data.

### Pre‐ and postnatal growth

A second cohort of pregnant rats were dosed with Dof or water (control *n* = 4, Dof *n* = 11) on GD 11–14 and then left undisturbed for the remainder of the gestation. At postnatal day 3, pups were weighed and litter sizes were reduced to a maximum of 8 to control for nutrition. Pups were reweighed at days 5, 10, 17, 24 and then weekly until 12 weeks age. At weaning, male offspring were separated from the dam and group housed until commencement of telemetry experiments.

### Telemetric measurement of blood pressure in adult offspring

At 3–6 months of age, male offspring (control *n* = 7, Dof *n* = 8) underwent surgical implantation of radiotelemetery blood pressure (BP) transmitters (PAC‐40, Data Sciences International, St. Paul, MN) as described previously (Furlong et al. 2014). Briefly, rats were anesthetized with a mixture of ketamine (60 mg/kg) and medetomidine (300 μg/kg), injected intraperitoneally. The anesthesia was deemed adequate when the animal did not exhibit a withdrawal reflex to nociceptive stimulation (a pinch of the hind paw). Once anesthetized, the rat was placed in a supine position and the abdomen shaved. The skin was sterilized with betadine and a midline incision was made through the abdominal skin and the abdominal muscles separated at the linea alba. The intestines were gently removed from the abdominal cavity to expose the aorta. All branches of the aorta between the right renal artery and the iliac bifurcation were temporarily occluded with sutures to vascularly isolate the abdominal aorta. The aorta was clamped proximally and distally with microvascular clamps and pierced just above the iliac bifurcation with a 21 gauge needle. The tip of the pressure sensing catheter was inserted and advanced approximately one centimeter proximally into the aorta. The site was sealed using n‐butyl cyanocryalate tissue adhesive and a cellulose patch to promote hemostasis and support fibrin growth. The intestines were carefully repositioned inside the abdominal cavity and the body of the transmitter was sutured to the inner surface of the abdominal muscles. The abdominal muscles were sutured back together and the skin incision was stapled closed.

### Postoperative care

Rats were returned to clean cages and placed on a heat mat to maintain body temperature throughout the initial recovery period. All animals were housed individually until the conclusion of experimentation to avoid risk of wound damage by littermates. Procaine penicillin (30 mg/kg), buprenorphine (0.01 mg/kg), the nonsteroidal antiflammatory agent carprofen (4 mg/kg), and glucose (2.5 ml 5% solution) were administered subcutaneously before anesthesia was reversed with atipamezole‐HCl (1 mg/kg, Antisedan, Pfizer) administered intraperitoneally. Rats were monitored closely for 3 h postsurgery, and twice daily thereafter. Twenty‐four hours after surgery, ibuprofen (15 mg/kg, Neurofen, Reckitt Benckiser) dissolved into sweetened jelly was supplied.

### Air jet stress protocol

Following a minimum of 1 week recovery, resting BP was measured over 24 h (scheduled sampling 5 min/h, ART software, DSI) before commencing an air jet stress (AJS) protocol (Furlong et al. 2014) that was repeated twice daily, 2 h apart, for 7 days, with BP recorded continuously on the first and last day. The AJS protocol comprised a 500 kPa jet of oxygen that was directed toward the face of the rat from a distance of 5–20 cm using an air gun. The protocol comprised an initial resting period of 30 min, followed by ~15 min of intermittent puffs of air. These puffs were broken up into nine blocks, each block made up of three puffs of air, 2 sec in duration and 10 sec apart. A 60 sec recovery time was allowed between each block, and analysis for the stress period was performed during these periods to avoid the inclusion of movement artifact. After the final block, rats were left undisturbed for a 90‐min recovery period before the protocol was repeated. This was performed every day from 12 pm for 7 days.

Following AJS, resting BP was recorded again over 24 h. At the conclusion of experiments, rats were euthanized by a lethal dose of pentobarbital sodium (120 mg/kg, Lethabarb, Virbac Animal Health, Fort Worth, TX) injected intraperitoneally. After the cessation of breathing and pulse, the heart was removed, fixed, and microdissected. No overt cardiac defects were observed in either group.

### Cardiovascular effects of Dofetilide on the adult

A separate cohort of telemetered male control rats were orally dosed with either water (*n* = 4) or Dof (2.5 mg/kg, *n* = 4). Rats were lightly anesthetized with isofluorane (2.5%) in room air, and water or Dof was administered by gavage. Immediately following dosing, rats were returned to their home cages and blood pressure was recorded for 5 h. Rats were left undisturbed for the duration of the recording. Averages of blood pressure and heart rate over 30 min were made every hour. The reported half‐life of Dof in the rat is approximately 30 min (Smith et al. 1992).

### Data analysis

Systolic, diastolic, and mean blood pressure and heart rate (HR) were calculated from the BP waveform post hoc on Spike 2 software (CED, Cambridge, UK) by a researcher blinded to group identity. Any regions containing large movement artifacts, arrhythmias, or signal drop‐outs were excluded from the analysis, and replaced by a section of equivalent duration immediately following the drop‐out.

Heart rate variability (HRV) and blood pressure variability (BPV) were determined in the frequency domain using FFT analysis of HR and systolic blood pressure (SBP) waveforms as described previously using a customized Spike2 (CED, UK) script (Parati et al. 1995). To avoid large peak artifacts near 0 Hz, the script uses a DC removal channel process with the time constant set at approximately 50% of the FFT duration. The frequency range for power spectra was set at 0–5.12 Hz thus setting the sample rate of the waveform channel to 10 Hz. The epoch duration used to create a power spectrum was set at 60 sec. The FFT size was manually set at 512 Hamming window, with 50% overlap and the resolution of power bands at 0.02 Hz. The frequency bands were defined as follows: very low frequency (VLF; 0–0.24 Hz), low frequency (LF; 0.24–0.74 Hz) and high frequency (HF; 0.74–3.2 Hz). In HRV, The VLF component is believed to reflect modifications by hormonal agents, the LF component to reflect cardiac sympathetic modulation and the HF component to reflect cardiac sympathetic modulation. In BPV, VLF is believed to reflect myogenic function (Stauss et al. 2008), LF to reflect sympathetic vasomotor modulation and HF to reflect either respiratory‐related hydraulic effects on venous return and stroke volume (Cerutti et al. 1994) or respiratory modulation of sympathetic vasomotor function (Menuet et al. 2017).

Spontaneous baroreflex sensitivity (sBRS) was derived from SBP and pulse interval (PI) using the sequence technique. Briefly, ramps of increasing or decreasing SBP over a minimum of four beats were identified, and plotted against the corresponding PI, with a delay of 3,4,5 beats. A positive linear regression with a correlation coefficient >0.8 was the criteria for inclusion, and sensitivity was indicated by the slope of the regression. Baroreflex effectiveness index (BEI), a measure of baroreflex recruitment, was determined by calculating the ratio of the number of sBRS sequences against the total number of SBP ramps (Di Rienzo et al. 2001).

All data are presented as mean ± SE. Embryonic HR and 24 h cardiovascular data were compared using an unpaired *t*‐test**.** Offspring weight and cardiovascular data from the telemetry studies were compared using two‐way ANOVA with repeated measures, and pair‐wise comparisons were made using a *t*‐test with Holm–Sidak correction for multiple comparisons. *P* < 0.05 was considered statistically significant.

## Results

### Acute effect of Dof on the embryo

**Figure 1:**
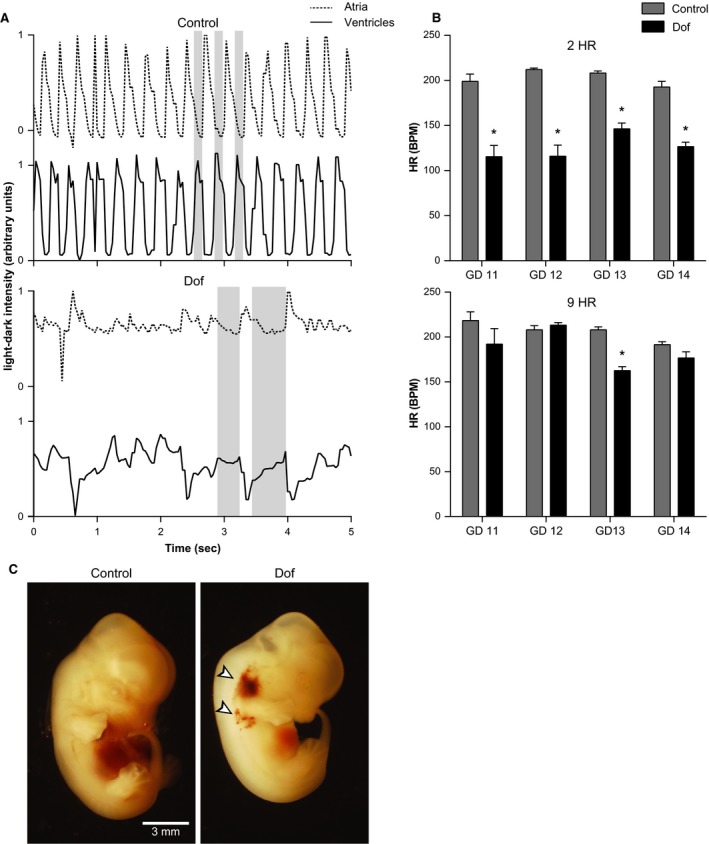
(A) Changes in light–dark intensity of the atria and ventricles identified by a video recording of beating embryonic hearts at GD13. The ordinate illustrates the cyclic fluctuation between systole (1) and diastole (0) in the atria (upper panels) and ventricles (lower panels). Gray bars indicate episodes of ventricular contraction. Two hours after dosing the dam with water (control embryo) a coordinated cardiac rhythm was evident, with ventricular systole occurring during atrial diastole. In contrast, after Dofetilide (Dof) treatment, the embryo's cardiac contractions were irregular and uncoordinated. (B) Mean HR after dosing on GD11–GD14. Two hours (upper panel) after dosing, the HR in the Dof‐exposed pups was ~40% slower than controls. By 9 h after dosing (lower panel), HR in the Dof‐exposed pups had mostly recovered, with the exception of GD13. (C) Examples of embryos (GD14) 2 h after dosing with water (left) or Dof (right). Note the hemorrhages at base of the forelimb (arrow heads) and lack of digital contours, digital ray and scalloping between digits. Dof; dofetilide, GD; gestational day, HR; heart rate. *n* = 8–14 embryos, 1–3 dams per time point.

Control GD13 embryo hearts exhibited regular, short‐lasting atrial and ventricular contractions that occurred in a coordinated and rhythmic pattern consistent with a normal cardiac cycle (Fig. 1A, upper panels). In contrast, Dof‐exposed embryos displayed prolonged, poorly coordinated atrial and ventricular systoles and missed beats (Fig. 1A, lower panels). At 2 hours after dosing, the average HR in Dof‐exposed embryos was ~40% slower than the control embryos on each gestational day (Fig. 1B, upper panel). At 9 h after dosing, HR was not different between groups, except for GD13 (Fig. 1B, lower panel). In the litter examined at GD14, after 4 days of Dof exposure, local hemorrhages and atypical limb development were observed in 7 of the 12 surviving embryos (Fig. 1C).

**Figure 2:**
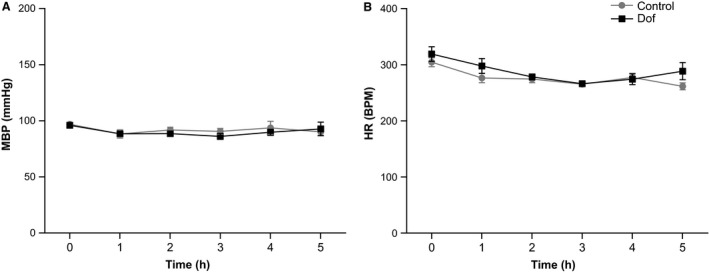
Mean BP (A) and HR (B) over 5 hours following oral administration of Dofetilide (Dof) (2.5 mg/kg) or water in the adult rat. In contrast to embryos, Dof had no effect on BP (*P* = 0.68) or HR (*P* = 0.13) in the adult rat. BP, blood pressure; HR, heart rate.

In a separate study, administration of the same dose of Dof (2.5 mg/kg) was found to have no effect on BP (*P* = 0.44) or HR (*P* = 0.14), compared to water (*n* = 4 per group), in adult male control rats (400–500 g) over a recording period of 5 h (Fig. 2).

### Postnatal growth

**Figure 3:**
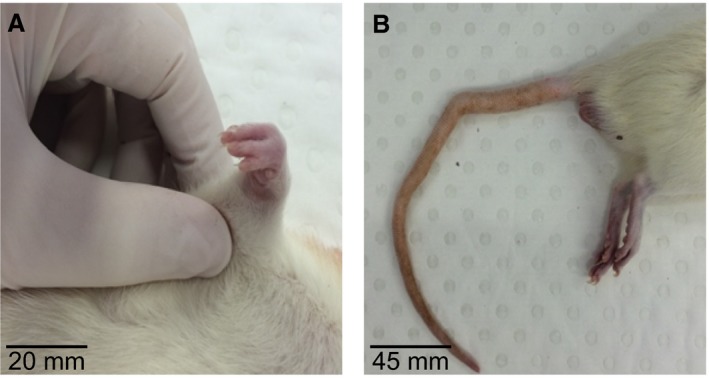
Examples of structural malformations observed in adult offspring following prenatal Dofetilide exposure. (A) shows fused digits in the forelimb in one rat. (B) shows kinks in the tail that was observed in two rats.

Of the 11 rats dosed with Dof for 4 days, six failed to litter, despite confirmation of mating by presence of sperm on vaginal swab. The average litter size of the five remaining dams was 6.4 ± 4.1 pups. In contrast, all four controls dams produced viable litters with a mean litter size of 14.3 ± 0.5 pups. Dof offspring were significantly lighter on postnatal day 3 compared to age‐matched controls (control 9.2 ± 0.2 g vs. Dof 8.3 ± 0.5 g; *P* = 0.02), and remained approximately 10% lighter than controls until the completion of experiments (3–6 months age, *P* < 0.01). In addition, structural malformations were observed in three adult offspring. One of the rats had a fused digit on the forelimb, and two rats had kinked tails (Fig. 3).

### Effects of Dof on adult offspring cardiovascular parameters at rest

**Figure 4:**
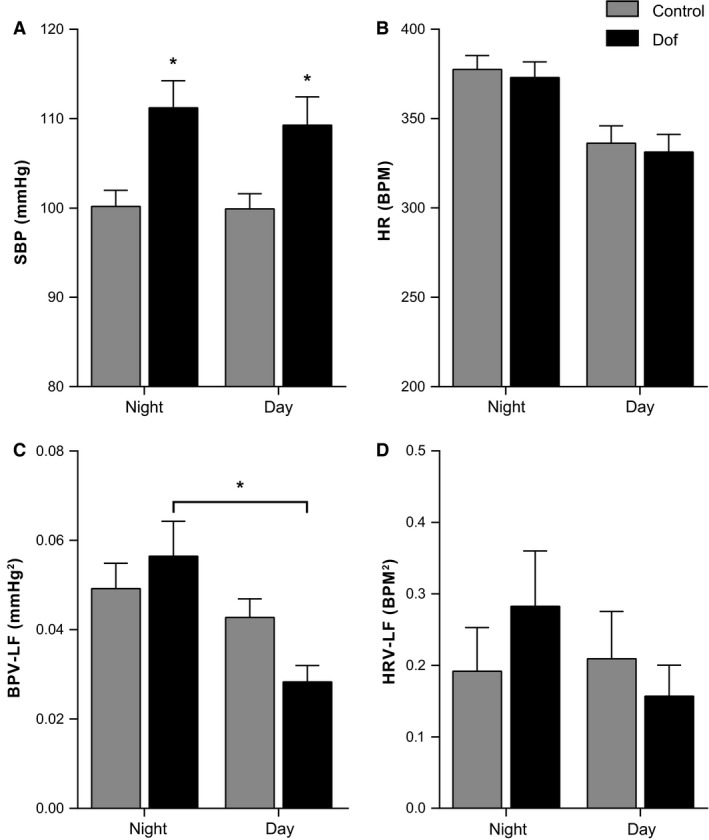
Baseline SBP, HR, and sympathetic indices of BPV‐LF and HRV‐LF in control and Dofetilide (Dof) rats during night and day. Arterial blood pressure was measured for 5‐min per hour for 24 h under resting conditions using radiotelemetry. SBP and HR and the variability were derived from the waveform post hoc. (A) SBP was significantly higher in Dof rats both at night (control 103.8 ± 3.9 vs. Dof 111.2 ± 3.0 mmHg, *P* = 0.01) and day (control 103.1 ± 3.5 vs. Dof 109 ± 3.2 mmHg, *P* = 0.03). (B) HR was not different between groups in either night or day. (C, D) There were no statistical differences between groups in the LF component of BPV or HRV. BPV, blood pressure variability; Dof; dofetilide, HR, heart rate; HRV, heart rate variability, LF; low frequency, SBP, systolic blood pressure. *n* = 7–8 rats.

BP was recorded in the conscious rat over 24 h to determine basal cardiovascular parameters (Table 1). Diastolic, mean, and systolic BP were all ~10 mmHg higher in Dof offspring both at night and day (Fig. 4A). HR was not significantly different between groups (Fig. 4B). BPV was not different between groups at night or day (Table 2), however, Dof rats displayed elevated LF variability at night compared to day (*P* = 0.03, Fig. 4C). A similar trend of diurnal variation was observed in HRV in the Dof offspring (*P* = 0.06, Fig 4D). Examination of the night:day ratio in LF BPV found Dof to be significantly higher than controls (control 1.2 ± 0.1, Dof 2.1 ± 0.3, *P* = 0.01). There were no differences in indices of baroreflex function between groups (Table 1).

**Table 1:** Comparison of resting cardiovascular parameters between control and Dofetilide (Dof) offspring

	Night			Day		
	Control	Dof	*P* value	Control	Dof	*P* value
SBP (mmHg)	100 ± 1.8	111 ± 3	**0.01**	100 ± 1.7	109 ± 3.2	**0.03**
MBP (mmHg)	83 ± 1.9	92 ± 2.3	**0.01**	82 ± 1.8	89 ± 2.4	**0.03**
DBP (mmHg)	70 ± 2.5	82 ± 3.7	**0.02**	67 ± 2.5	80 ± 4	**0.02**
HR (bpm)	380 ± 8.8	370 ± 8.7	0.71	339 ± 10.9	331 ± 9.9	0.72
sBRS+ (ms/mmHg)	1.3 ± 0.1	1.4 ± 0.1	0.71	1.4 ± 0.1	1.6 ± 0.2	0.11
sBRS– (ms/mmHg)	1.2 ± 0.1	1.3 ± 0.1	0.44	1.2 ± 0.1	1.4 ± 0.2	0.84
BEI	0.2 ± 0.02	0.2 ± 0.01	0.93	0.2 ± 0.02	0.2 ± 0.01	0.73

Mean ± SE of cardiovascular parameters at night and day. *P* values highlighted in bold indicate a statistically significant difference between groups. *P* value from unpaired *t*‐test. BEI, baroreflex effectiveness index; DBP, diastolic blood pressure; HR, heart rate; MBP, mean blood pressure, SBP, systolic blood pressure, sBRS+, spontaneous baroreflex sensitivity from pressor ramps, sBRS‐, spontaneous baroreflex sensitivity from depressor ramps.

**Table 2:** Comparison of resting heart rate and blood pressure variability

		Night			Day		
		Control	Dof	*P* value	Control	Dof	*P* value
HRV (bpm^2^)	VLF	3.30 ± 0.37	3.70 ± 0.62	0.58	2.46 ± 0.20	2.48 ± 0.39	0.97
	LF	0.19 ± 0.06	0.28 ± 0.08	0.38	0.21 ± 0.07	0.16 ± 0.04	0.52
	HF	0.84 ± 0.16	0.89 ± 0.13	0.80	0.67 ± 0.21	0.50 ± 0.07	0.41
	LF:HF	0.66 ± 0.25	0.29 ± 0.05	0.16	0.28 ± 0.02	0.27 ± 0.05	0.87
	TPWR	2.80 ± 0.79	4.47 ± 0.77	0.16	3.34 ± 41	2.85 ± 0.47	0.45
BPV (mmHg^2^)	VLF	0.28 ± 0.03	1.20 ± 0.85	0.30	0.28 ± 0.03	0.74 ± 0.50	0.38
	LF	0.05 ± 0.01	0.06 ± 0.01	0.46	0.04 ± 0.004	0.03 ± 0.003	**0.03**
	HF	0.04 ± 0.005	0.28 ± 0.23	0.20	0.04 ± 0.01	0.14 ± 0.12	0.58
	LF:HF	1.27 ± 0.01	1.42 ± 0.20	0.42	1.14 ± 0.05	1.03 ± 0.14	0.56
	TPWR	0.36 ± 0.03	1.64 ± 1.18	0.31	0.36 ± 0.03	0.95 ± 0.66	0.39

Mean ± SE of heart rate and blood pressure variability at night and day. *P* values highlighted in bold indicate a statistically significant difference between groups. *P* value from unpaired *t*‐test. Dof, Dofetilide; BPV, blood pressure variability; HF, high frequency; HRV, heart rate variability; LF, low frequency; LF:HF, low‐frequency to high‐frequency ratio; TPWR, total power; VLF, very low frequency.

### Effects of Dof on cardiovascular responses to air jet stress

**Figure 5:**
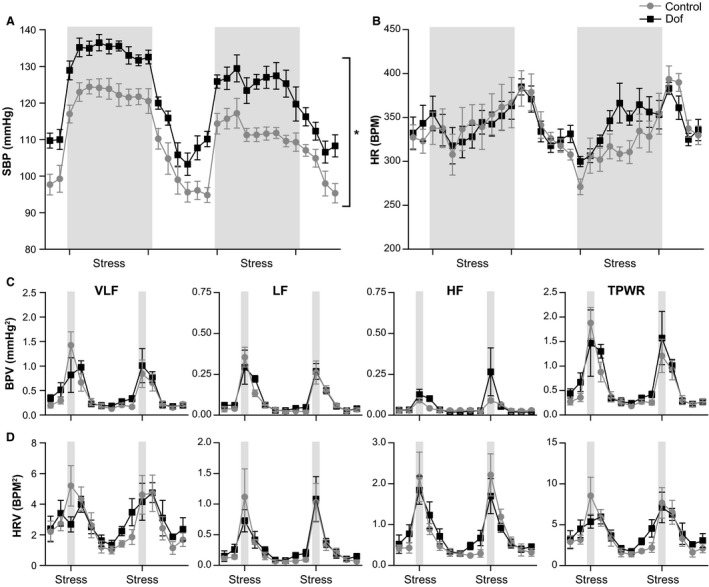
(A) Changes in SBP during Day 1 of AJS. The protocol consisted of 30‐min baseline, followed by nine blocks of three air puffs (gray bars), repeated every 60 s, and a 90‐min recovery period after which the protocol was repeated. Data were measured every 15 min during the baseline and recovery periods, and every 90 s during AJS. Measurements during AJS were obtained from the 60‐s intervals between blocks of puffs to avoid the inclusion of movement artifacts in the analyses. SBP was significantly higher throughout the protocol in Dof (*P* = 0.002), however, there was no difference in the change in SBP in response to AJS. (B) The HR response to AJS was variable, although there was a significant increase over baseline. There was no difference in the HR response between groups. Similarly, BPV (C) and HRV (D) increased in response to AJS, with no difference between groups. AJS; air jet stress, BPV; blood pressure variability, Dof; dofetilide, HR; heart rate; HRV; heart rate variability, SBP; systolic blood pressure. *n* = 7–8 rats.

Rats were exposed to 15‐min AJS twice daily for 7 days to determine both the acute cardiovascular responses to psychological stress, and to assess any alterations in the response to repeated psychological challenges. The AJS protocol consisted of intermittent puffs of air directed at the rat's face. On day 1, SBP was significantly higher in Dof rats during the baseline recording prior to the AJS protocol, consistent with the 24 h baseline data (Fig. 5A). The magnitude of the pressor response to AJS, however, was similar between groups (control +17.7 ± 3.4 vs. Dof +18.91 ± 0.9 mmHg, *P* = 0.78), as was the rate of recovery to baseline at completion of the protocol (Fig. 5A). The pressor response to the second AJS (2 h later) was reduced in control and Dof rats by approximately 40% (*P* < 0.01 for control and Dof). The HR response to AJS was also similar between groups, with HR increasing modestly over the course of the AJS and recovering to baseline levels within 30 min (Fig. 5B). AJS evoked a large increase in BPV (Fig. 5C) and HRV (Fig. 5D) in all frequency bands. No clear effects of AJS on sBRS or BEI were observed in either control or Dof rats.

### Attenuation of blood pressure response following a week of repeated air jet stress

**Figure 6:**
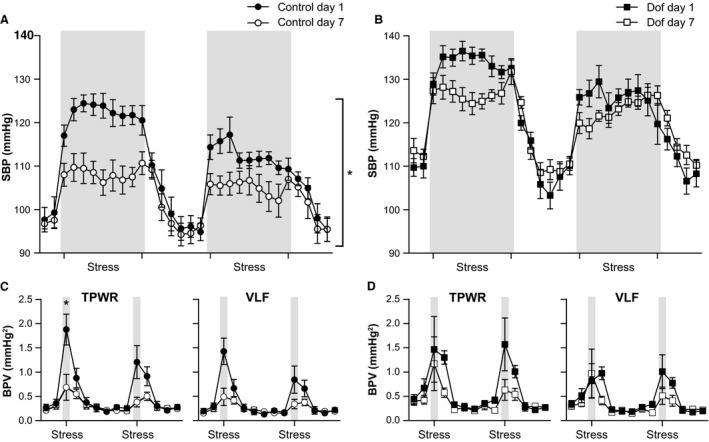
Comparison of changes in SBP following exposure to AJS on day 1 vs. day 7 in control (A) and Dofetilide (Dof) offspring (B), after repeated daily AJS for 7 days. Baseline SBP was unchanged in both groups. In controls, the SBP pressor response was significantly attenuated after 7 days of repeated AJS (*P* = 0.0001) (A). This attenuation was greatly reduced in Dof rats, where there was no significant difference between Day 1 and Day 7 (*P* = 0.48) (B). A similar trend in attenuation was observed in TPWR and VLF spectra in BPV in controls (C), but not Dof (D). AJS; air jet stress, BPV; blood pressure variability, Dof; dofetilide, SBP; systolic blood pressure; VLF; very low frequency, TPWR; total power. *n* = 7–8 rats.

The AJS protocol was repeated daily for 7 days to assess long‐term adaptation to stress. Similar to day 1, SBP remained higher on day 7 in Dof rats throughout the protocol. The stress‐evoked increase in SBP on day 7 was not significantly different between groups (control +10.4 ± 1.2 mmHg, Dof +15.0 ± 3 mmHg, *P* = 0.35). However, a significant attenuation of the pressor response to AJS was observed in controls on Day 7 compared to Day 1 (*P* = 0.0001, Fig. 6A), that was not seen in the Dof group (*P* = 0.48, Fig. 6B). This attenuation in response was also reflected in the total power and VLF of BPV (Fig. 6C vs. 6D).

### Resting BP following a week of AJS

At the completion of the 7‐day AJS protocol, BP was recorded for another 24 h. Dof offspring showed a small but significant increase in SBP at night (night, +3.9 ± 1.7 mmHg, *P* = 0.05; day, +2.8 ± 2.0 mmHg, *P* = 0.2), whereas HR was reduced during the day (day, −18 ± 5 bpm, *P* = 0.01; night, −7 ± 4 bpm, *P* = 0.15). There was no change in SBP (night, +1.1 ± 0.7 mmHg, *P* = 0.20; day, +0.24 ± 0.84 mmHg, *P* = 0.79) or HR (day, −11 ± 8 bpm, *P* = 0.26; night, 0 ± 6 bpm, *P* = 0.98) in control offspring.

## Discussion

The main finding of this study was that Dof‐induced bradycardia in rat embryos from GD 11–14 programmed mild, but persistent hypertension at 3–6 months of age, accompanied by a blunted adaptation of the cardiovascular response to repeated psychological stress. Abnormal cardiovascular responses to stress are a sign of autonomic dysregulation and are linked to increased incidence of hypertension in the long term (al'Absi and Arnett 2000; Dimsdale 2008), suggesting a potential pathophysiological mechanism for hypertension in these animals. Our earlier studies showed that a similar degree of embryonic bradycardia was associated with embryonic hypoxia (Ritchie et al. 2013, 2015). We therefore propose that embryonic hypoxia (or its consequences) may be the underlying programming stimulus in these offspring.

### Dof induces severe bradycardia and arrhythmia in the embryo for up to 8 h

Embryos exposed to 2.5 mg/kg Dof from GD 11–14 suffered a dramatic drop in HR (−40%) and severe arrhythmia suggestive of atrioventricular blockade, although cardiac rhythm could not be analyzed using the present methodology and would require examination of embryonic ECGs. In contrast, no effect on HR was observed in adult male rats exposed to the same dose, consistent with previous studies (Ritchie et al. 2015). In the rat, the myocardial action potential relies on the *I*_Kr_ channel only from GD 10, when the embryonic heart begins beating, until GD 14, when other channels dominate for the duration of the animal's life (Davies et al. 1996; Nilsson et al. 2013; Ritchie et al. 2013). Thus, there is a 5‐day period during gestation when the embryo is sensitive to *I*_Kr_ blockade with Dof. This period also coincides with embryonic organogenesis and therefore, susceptibility to teratogen‐induced malformation. Even very high doses of Dof (up to 88 mg/kg) administered either immediately before (GD 9) or after (GD 15) this period do not have any teratogenic or embryotoxic effects, suggesting that the teratogenicity of Dof is conferred through the effect on heart rate (Webster et al. 1996; Abela et al. 2010).

The shift in dependence to other channels also explains the resistance to the bradyarrhythmic effects of *I*_Kr_ blockade in the adult rat, consistent with other reports (Abrahamsson et al. 1994; Danielsson et al. 2007; Abela et al. 2010). In this study, while no effect on heart rate or blood pressure was observed, we acknowledge that a limitation of this result was the small sample size. To further define the effects of gestational Dof on cardiovascular variables, telemetry studies would need to be performed on pregnant dams. However, considering the known mechanism of action of Dof (Danielsson et al. 2007), and taken together with previous studies on adult resistance to Dof (Abrahamsson et al. 1994; Webster et al. 1996), we believe that effects of daily Dof administration are confined to the embryo, and that maternal cardiovascular function is likely to be unaffected at the dose tested.

### The effect of chronic low dose Dof exposure on perinatal development and survival

By GD 14, 4 days of repeated low dose Dof exposure had induced limb hemorrhages in embryos, and one of the Dof offspring in the adult study had a fused digit malformation. Limb hemorrhages and subsequent digit malformations are a well‐described side effect of embryonic exposure to *I*_Kr_ blockers such as Dof (Webster et al. 1996; Ritchie et al. 2013). While orofacial and cardiovascular defects were not observed in this study, they have been observed following higher doses of Dof (Webster et al. 1996). Interestingly, intermittent interruptions to the embryonic circulation, achieved by clamping the uterine artery, induce identical stage‐specific malformations in the embryo (Lawler et al. 1981; Webster and Abela 2007). Limb hemorrhages can be caused by a hypoxia‐induced overexpression of vascular endothelial growth factor (VEGF) that degrades the basement membrane and alters vascular permeability (Lee et al. 2001; Nanka et al. 2006; Ream et al. 2008). Unpublished data from this laboratory showed a single dose of Dof (5 mg/kg) at GD13 increased VEGF expression for 6–8 h after dosing, preceding the appearance of edema and hemorrhages in the limbs by 6–12 h (Sood S. et al., unpubl. data). This was not examined in this study, and should be the focus of future studies examining potential VEGF overexpression and its role preceding embryonic limb malformation. Taken together, these observations suggest that the teratogenic effects of Dof may be due to the embryos’ overcompensatory responses to tissue hypoxia.

Over half of the Dof‐treated dams failed to litter. In the remaining dams, litter sizes were much lower than controls, indicating higher rates of fetal resorptions, and neonatal mortality was higher. This demonstrates the adverse impact of Dof on pre‐ and postnatal survival, even at doses lower than those that used in previous studies (Ritchie et al. 2013). These are also commonly reported features of hypoxia during pregnancy (Ream et al. 2008; Bourque et al. 2013; Jang et al. 2015), but not maternal undernutrition. Dof pups were also growth restricted, a common characteristic in programmed offspring (Barker 1995). The exact cause of fetal growth restriction in our study remains uncertain, but could be linked to the bradycardic effect on tissue perfusion, and subsequent hypoxia and/or undernutrition. Since a maternal low oxygen environment is known to reduce maternal food consumption (de Grauw et al. 1986; Ream et al. 2008) it has been suggested that undernutrition rather than hypoxia may be responsible for the growth restriction observed in programming models using hypoxia as the insult. However, 24 h of maternal oxygen depletion (8% O_2_) acutely reduces fetal growth, whereas the same period of total food deprivation has no effect (Ream et al. 2008), indicating that the embryos are more sensitive to reductions in oxygen than nutrients. Moreover, hypoxia‐induced growth restriction has also been reported in chick embryos, where maternal nutrition is not a factor (Sharma et al. 2006). Hypoxia is believed to reduce growth by shifting the fetal metabolism to favor anaerobic glycolysis (Tapanainen et al. 1994). Thus, taking into consideration the adverse effect of Dof on the incidence of fetal hemorrhages, structural malformations, litter viability, and fetal growth, all of these consequences may be better explained by the effects of a bradycardia‐induced hypoxia rather than reduced nutrient delivery. However, future studies should examine whether Dof treatment affects maternal food consumption and contributes to the phenotype observed.

### Effects of gestational Dof on adult blood pressure and autonomic function at rest

This is the first study to show that administration of Dof to the pregnant dam produces raised resting BP in the adult offspring. While this study only examined male offspring, future studies on the effects of gestational Dof on female offspring will be valuable in determining the gender‐specific risk profile of the drug.

The mechanism by which Dof programs hypertension is uncertain, but may also involve fetal hypoxia, as described above. Comparable findings have been reported in models of hypoxia‐programmed hypertension using maternal exposure to a low (10–12%) oxygen environment (Bourque et al. 2013; Rook et al. 2014; Svitok et al. 2016). One study only found differences in BP in response to stress (Peyronnet et al. 2002), however, this is a well‐reported feature of prehypertension, suggesting that these offspring may have developed hypertension at a later age (Matthews et al. 2004). Thus, we propose that the developmental, teratogenic, and programming effects of prenatal Dof may all be consequences of embryonic hypoxia.

A previous study reported that programmed hypertension following maternal hypoxia was associated with changes in autonomic function, including an increased LF:HF ratio in HRV during the day and reduced baroreflex sensitivity at night (Svitok et al. 2016). The investigators suggested that even subtle changes such as these may contribute to the development of hypertension in this model. Although this study did not find differences in HRV or baroreflex sensitivity, the LF component in BPV during the day (inactive phase) was lower in Dof than controls. This difference was also reflected in the increased night:day ratio in this variable, and suggests that diurnal fluctuations in sympathetic modulation of SBP may be exaggerated in Dof offspring. However, it must be noted that noninvasive measures of autonomic function can be highly variable, and results should be interpreted with caution (Heathers 2014). Further evidence for this hypothesis arises from a study in which direct measures of autonomic modulation, such as muscle sympathetic nerve recordings, showed that hypoxia‐programmed offspring exhibit elevated sympathetic activity compared to their normoxic counterparts (Rook et al. 2014). Moreover, this sympathetic overactivity is present in these animals prior to the development of hypertension, which takes several months to manifest. Taken together, these studies suggest that autonomic dysfunction may underlie the development of hypertension in hypoxia‐programmed offspring. Further evidence is required to confirm that elevated sympathetic activity underlies Dof‐programmed hypertension.

### Effects of gestational Dof on the cardiovascular response to air jet stress

There is growing support for the idea that an exaggerated response to psychological stress may underlie the increase in blood pressure observed in programmed hypertension (Vallee et al. 1997; Peyronnet et al. 2002; Esler et al. 2008; O'Regan et al. 2008). There are a number of ways in which an exaggerated stress response may present in programmed offspring. For example, numerous studies have reported that programmed offspring exhibit a larger increase in blood pressure or heart rate when stressed (Tonkiss et al. 1998; O'Regan et al. 2008) as well as delayed recovery to baseline (Igosheva et al. 2004; O'Regan et al. 2008). In this study, exposure to AJS‐evoked cardiovascular responses of a similar magnitude and recovery time. However, since the evoked response was not diminished in the Dof group despite a higher resting blood pressure, this suggests that SBP was regulated around a higher set‐point, whereas the range, sensitivity, and duration of the SBP response to stress remained unchanged. Such exposure to higher levels of BP, even acutely, poses cardiovascular risk. For example, for every 20 mmHg increase in blood pressure, the risk of mortality from stroke and ischemic heart disease is doubled (Jones and Hall 2004), whereas acute stressful events are well reported to increase the risk of sudden death (Wilbert‐Lampen et al. 2008). Thus, although Dof offspring did not show an exaggerated response, the higher BP response in absolute terms nevertheless poses a greater risk for short‐term cardiovascular events.

An exaggerated stress response may also present as impaired habituation to a previously encountered stressor. Both Dof and control offspring showed normal short‐term habituation, with blunted SBP responses to a second AJS on day 1 (McEwen 1998; Grissom and Bhatnagar 2009). However, differences in the ability to habituate to repeated stress emerged after 7 days. In controls, the SBP response to daily AJS had diminished significantly – a feature of allostasis that is well described (Fuchs et al. 1998; McEwen 1998; Bechtold et al. 2009). This habituation was significantly impaired in Dof offspring. The process of habituation to repeated stress is known to be dependent on negative feedback regulation of glucocorticoids within the hippocampus and subsequent modulation of the hypothalamic‐pituitary‐adrenal (HPA) axis (McEwen 2007; Tasker and Herman 2011; Herman 2013). Decreased glucocorticoid receptor expression and reduced HPA axis feedback has been reported in models of hypoxia‐programmed hypertension (Fan et al. 2009; Gonzalez‐Rodriguez et al. 2014).

Interestingly, habituation to repeat psychological stress is also impaired in the Borderline Hypertensive rat (BHR); a model with genetic predisposition to hypertension (Bechtold et al. 2009). The lack of attenuation appears to be accompanied by differences in the sensitivity to glucocorticoids, highlighted by the inconsistent effects of glucocorticoid receptor blockade in the BHR versus normotensive strains (Bechtold et al. 2009; Scheuer 2010). While the mechanisms remain unclear, aberrant actions of glucocorticoids and/or their feedback may underlie the exaggerated responses to stress in BHRs and Dof rats. Since cardiovascular reactivity to chronic, psychological stress is an important factor in the development of hypertension (Esler et al. 2008), it is possible that inadequate adaptation to environmental stress contributes to the hypertension observed in Dof offspring and may worsen over time.

After 7 days of AJS, baseline SBP was raised slightly in Dof, but not control offspring at night, suggesting that repeated exposure to stress may elevate resting blood pressure. Although the observed increase in BP was modest, this is still a dramatic effect considering that the stimulus strength and duration used in this study was mild. Studies that have reported elevations in baseline BP following chronic stress, even in susceptible strains like the BHR, impose weeks to months of stress exposure and usually involve moderate‐severe stressors such as electric shock or restraint paradigms (Lawler et al. 1981; Mansi and Drolet 1997; Bechtold et al. 2009; Xiao et al. 2013). It therefore seems likely that the allostatic load evoked by chronic stress exposure over weeks to months would exacerbate the resting hypertension in this model. The latter possibility seems reasonable, since the physiological mediators that are associated with stress adaptation also play a role in the pathophysiology that occurs when there is sustained activation of the stress response (McEwen 2000).

### Potential mechanisms of Dof‐induced programming

A previous study from this lab has shown that higher doses of Dof (5 mg/kg) reduced embryonic heart rate by ~50% and produced tissue hypoxia in the limbs of treated embryos (Ritchie et al. 2013). This was assessed with pimonidazole hydrochloride, a marker that produces adducts at a tissue PO_2_ of 10 mmHg or lower (Raleigh et al. 1999). The period of hypoxia was shown to coincide with the period of Dof‐induced bradyarrythmia, suggesting that the oxygenation of the embryo parallels embryonic heart rate (Ritchie et al. 2013). Furthermore, a study on almokalant, another *I*_Kr_ channel blocker, achieved the same degree of bradycardia as in this study (~40% on GD 11) and observed intense systemic pimonidazole staining in drug‐exposed embryos compared to controls (Danielsson et al. 2003). Based on these results, we hypothesize that the degree of bradycardia achieved in this study was comparable and therefore capable of producing embryonic hypoxia.

Some studies have suggested that the teratogenic effects of prolonged embryonic bradycardia are related to ischemia/reperfusion injury, rather than tissue hypoxia itself. The incidence of malformations was greatly reduced when pregnant rodents were pretreated with the oxygen free radical scavenger α‐phenyl‐N‐tert‐butyl‐nitrone before dosing with the *I*_*Kr*_ blocking agents, dimethadione, an antiepileptic drug, and almokalant (Wellfelt et al. 1999; Azarbayjani and Danielsson 2002). This suggests that oxidative stress may play a role in inducing embryonic malformations. Whether reactive oxygen species also play a role in the programming of cardiovascular disease is not well understood, but the programming effects of a hypoxic maternal environment have also been attenuated by antioxidant pretreatment (Giussani et al. 2012). Interestingly, oxidative stress is also common feature in many other programming models, including maternal undernutrition, maternal overnutrition, maternal smoking, (Luo et al. 2006) and maternal anemia (Grune et al. 2000). Finally, in addition to inducing hypoxia, Dof‐induced bradycardia would also increase tissue CO_2_ and promote acidosis, however, to our knowledge, there are few teratogenic or programming studies that have investigated this. Future studies will be valuable in discriminating between the role of hypoxia and acidosis on the embryonic effects of Dof.

## Conclusions

Fetal bradycardia and arrhythmia are well‐known obstetric risks associated with severe acute consequences. In this study, we demonstrate for the first time that relatively short episodes of pharmacologically induced bradycardia/arrhythmia programs cardiovascular dysfunction in the offspring. The causes may be due to aberrant autonomic responses to psychological stress, however, the neurobiological framework underpinning these responses remain to be determined.

The data also have implications for the use of cardioactive drugs during pregnancy. In humans, the *I*_Kr_ channel is present in both adult and embryo. Dof continues to be used in the USA for treatment of atrial arrhythmias and, despite its known teratogenic risks, is still prescribed to pregnant mothers in low dose where the benefit to the patient justifies the risk to her offspring (Joglar and Page 2014). This study highlights that in addition to the previously reported acute effects observed at higher doses, the long‐term consequences of low dose Dof use in pregnancy, and the potential increased cardiovascular risk to adult offspring, should also be considered. Further studies will be needed to test the long‐term effects of other class III antiarrhythmic drugs that are used commonly in other regions such as Europe and Australia, including sotalol and amiodorone (Cordina and McGuire 2010; Regitz‐Zagrosek et al. 2011). Finally, this study may have implications for the health of the offspring following the maternal use of other drugs that are known to affect the *I*_Kr_ channel.

## Conflict of Interest

None declared.

## Data Accessibility

## Supporting information




**Video S1.** Control Embryonic heart beat.

**Video S2.** Dof Embryonic heart beat.
